# The Effect of Foot Rehabilitation Protocol in Adults With Congenital Deformity of Foot: An Analytical Case Series

**DOI:** 10.7759/cureus.59102

**Published:** 2024-04-26

**Authors:** H. V. Sharath, Siddhi G Rathi, Pradhyum D Kolhe

**Affiliations:** 1 Department of Pediatric Physiotherapy, Ravi Nair Physiotherapy College, Datta Meghe Institute of Higher Education and Research (Deemed to Be University), Wardha, IND

**Keywords:** rehabilitation, physical therapy, flat foot, pes cavus, pes planus

## Abstract

Congenital deformities of the foot significantly challenge the mobility and quality of life of affected individuals. While surgical interventions are common, rehabilitation protocols tailored to address the specific needs of adults with congenital foot deformities are less explored. This case series aims to evaluate the effectiveness of a specialized foot rehabilitation protocol in improving functional outcomes and quality of life in adults with congenital foot deformities. A series of cases involving adults diagnosed with congenital foot deformities were enrolled in a structured rehabilitation program. The protocol incorporated a combination of therapeutic exercises, manual therapy, gait training, and orthotic management tailored to individual needs. Outcome measures included functional assessments, gait analysis, pain levels, and patient-reported outcomes at baseline, midpoint, and endpoint of the rehabilitation program. Preliminary findings from the case series indicate significant improvements in various outcome measures following the foot rehabilitation protocol. Participants demonstrated enhanced gait parameters, reduced pain levels, increased range of motion, and improved functional capacity. Moreover, subjective assessments revealed enhanced satisfaction and perceived improvements in quality of life among participants. The findings suggest that a tailored foot rehabilitation protocol can be beneficial in improving functional outcomes and quality of life in adults with congenital foot deformities. This underscores the importance of integrating comprehensive rehabilitation strategies alongside surgical interventions to optimize long-term outcomes and enhance the overall well-being of individuals with congenital foot deformities. Further research with larger sample sizes and controlled study designs is warranted to validate these findings and establish evidence-based rehabilitation guidelines for this population.

## Introduction

The foot's arch displays two extremes of anatomical structural position: the flat arch typical of the pes cavus and the high arch characteristic of the planus pes. Despite the fact that the foot is supported by three different arches, the medial longitudinal arch (MLA) has been identified as the arch with clinical significance in both of these illnesses [1,2]. In the past, flat feet were divided into two different categories: flexible and rigid. Surgery to realign the skeletal structure is frequently used to cure inflexible flat feet. Clinically asymptomatic cases of flexible flat feet do not need intervention [3].

Previous research indicates that 25% of the general population is thought to have flat feet. This prevalence seems to be higher in females and in people with wide feet and higher body mass indexes (BMIs). The frequency of flatfeet dramatically declines with age; at 3 years old, it is 54%, at 6 years old, it is 24%, and at 18 years old, it is 11.25% [4]. Pes cavus, which is characterized as having a high-arched foot, is a common pediatric foot variant that presents no symptoms. Nonetheless, some types of pes cavus, such as pes cavovarus (PCV) or pes calcaneocavus, might be symptomatic and linked to neurological disease and its accompanying muscular imbalance [5]. Its prevalence appears to rise with age, according to reports, from 2% at age 3 to up to 7% at age 16 [6]. However, the incidence, which ranges from 10.5% to 25% in adults, may be substantially higher [7,8].

Results point to the possibility that aberrant biomechanical factors associated with pes cavus (high arch) and pes planus (flat foot) may predispose a person to injury. For instance, planus feet move more during gait than normal feet do, and as a result, soft tissue injuries that interfere with this motion may occur. On the other hand, it is believed that cavus feet move less than planus feet [9,10]. The goal of the study is to look into how persons with congenital foot deformities respond to a foot rehabilitation program. The subject of the case series will be a person with a congenital deformity, which encompasses both pes cavus and pes planus. The foot rehabilitation protocol includes an exercise program. The study seeks to assess improvements in the MLA of the foot. Through investigating the possible advantages of an all-encompassing method, the study seeks to provide important information for the creation of successful treatment plans for patients with congenital deformities, such as pes cavus and pes planus.

## Case presentation

Case 1

**Figure 1:**
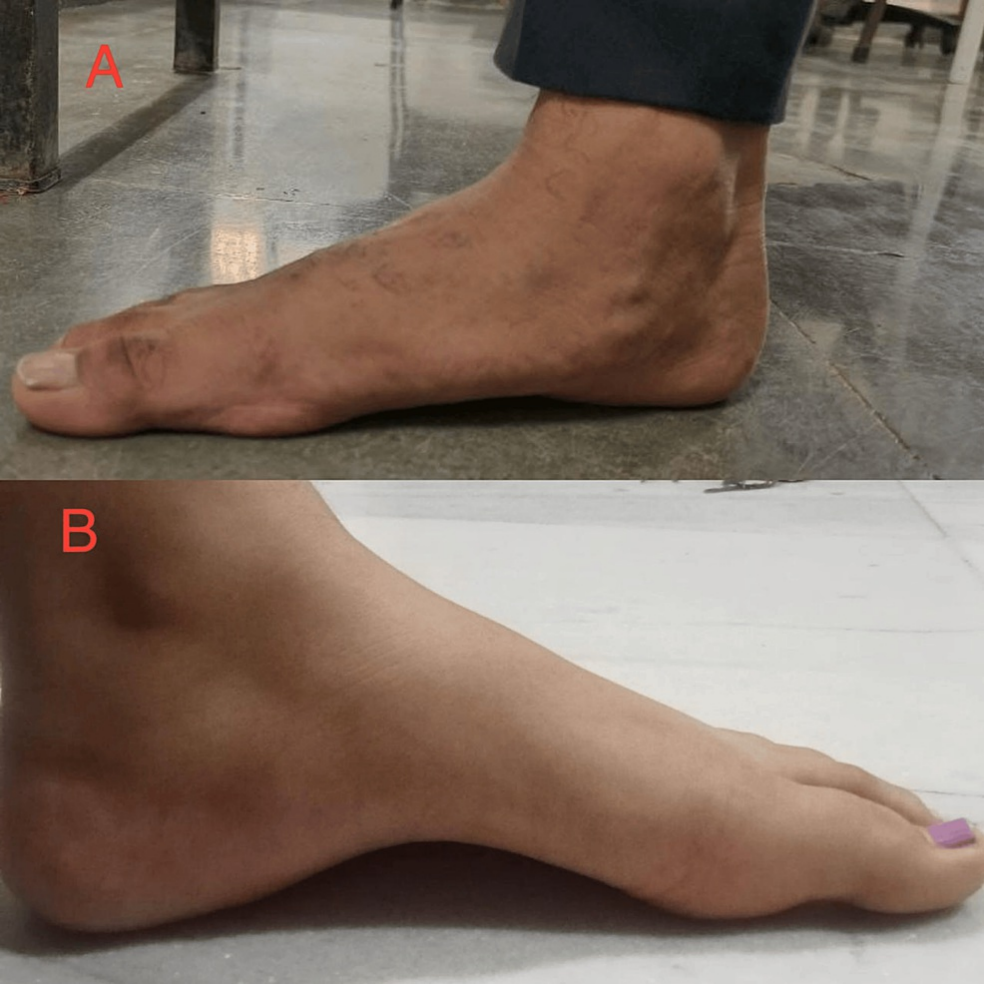
A: Flat foot; B: High-arched foot

A 19-year-old male presented to our musculoskeletal department of physiotherapy with the chief complaint of impaired gait due to congenital flatfeet. He is able to maintain regular activity levels, and his general examination reveals no anomalies. Clinical examination confirms that he has reduced arches in both feet, bilateral flatfeet. He never underwent any form of therapy to help his flat feet heal (Figure 1A). Before receiving rehabilitation, the patient with flat feet underwent manual muscle testing (MMT). Compared to plantarflexion and eversion, there was less strength in the dorsiflexion and inversion motions, which may be a sign of muscle imbalances related to the condition. During intense activity, the patient additionally complained of pain and unbalance. A visual analog scale is used to measure the patient's pain during physically demanding tasks. To look at and evaluate gait patterns, X-sens gait analysis is used in addition to foot assessment scales like foot posture index (FPI), foot function index (FFI), and foot and ankle ability measures (FAAM). Other than these foot abnormalities, no other noteworthy anomalies are observed. His lower limbs exhibit normal muscular tone and strength in terms of neurology. His prognosis for retaining functional independence is still favorable.

Case 2

A 20-year-old female presented to our musculoskeletal department of physiotherapy is experiencing pain due to congenital pes cavus. She is able to maintain regular activity levels, and her general checks reveal no anomalies. Her main complaints are heel inward tilt and soreness when walking long distances. It was discovered during the clinical evaluation that she has bilateral high arches (Figure 1B). A patient reports ankle instability and plantar fascia stiffness-related pain. An ankle sprain risk may be elevated by ankle instability. The patient did not receive any rehabilitation for their high arch feet. There was no prior history of neurological symptoms such as numbness, trauma, or resting discomfort. Her strength was assessed using MMT, which was done prior to her therapy. It was noted that the strength had decreased. In addition, foot assessment scales include the FPI, FFI, and FAAM. Examining and analyzing gait patterns is done using X-sens gait analysis. The degree of pain is measured using a visual analog scale. Her chances of maintaining her functional independence are quite good.

Physiotherapy management 

Physiotherapy management is involved in enhancing physical well-being and addressing specific conditions or injuries. A key component of this management includes tailored exercises aimed at improving strength, flexibility, and mobility. For instance, one exercise involves scrunching a towel with their toes for 15 minutes daily, promoting dexterity and strength in the feet and lower limbs. Additionally, targeted stretching routines play a crucial role in physiotherapy. Heel cord stretching, performed by standing with one leg forward and bending slightly at the knee while pressing the hips gently toward a wall, aids in improving flexibility and relieving tension in the lower extremities. This exercise, repeated 10 times with 30-second holds, fosters better alignment and mobility.

**Figure 2:**
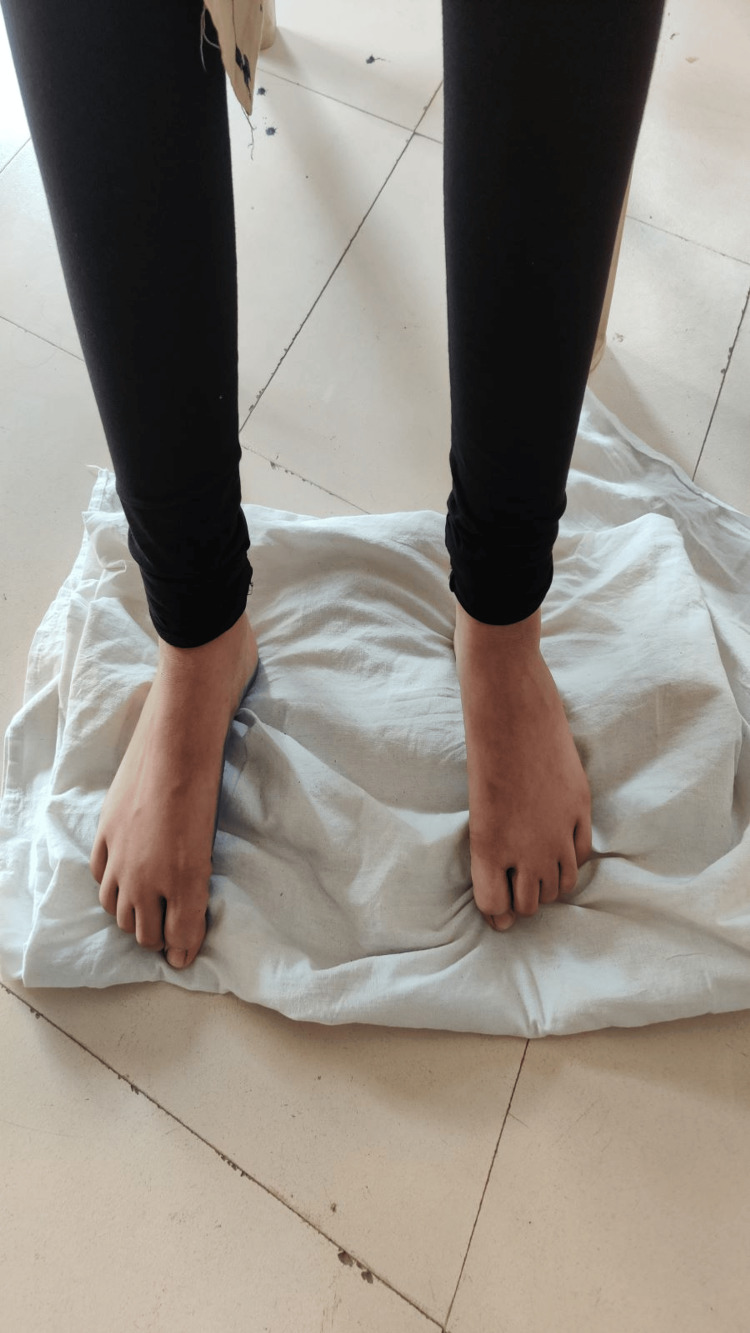
Towel scrunching exercise Instructing the individual to engage in the towel-gathering activity by scrunching a towel placed on the floor with their toes, dedicating 15 minutes to this exercise daily.

**Figure 3:**
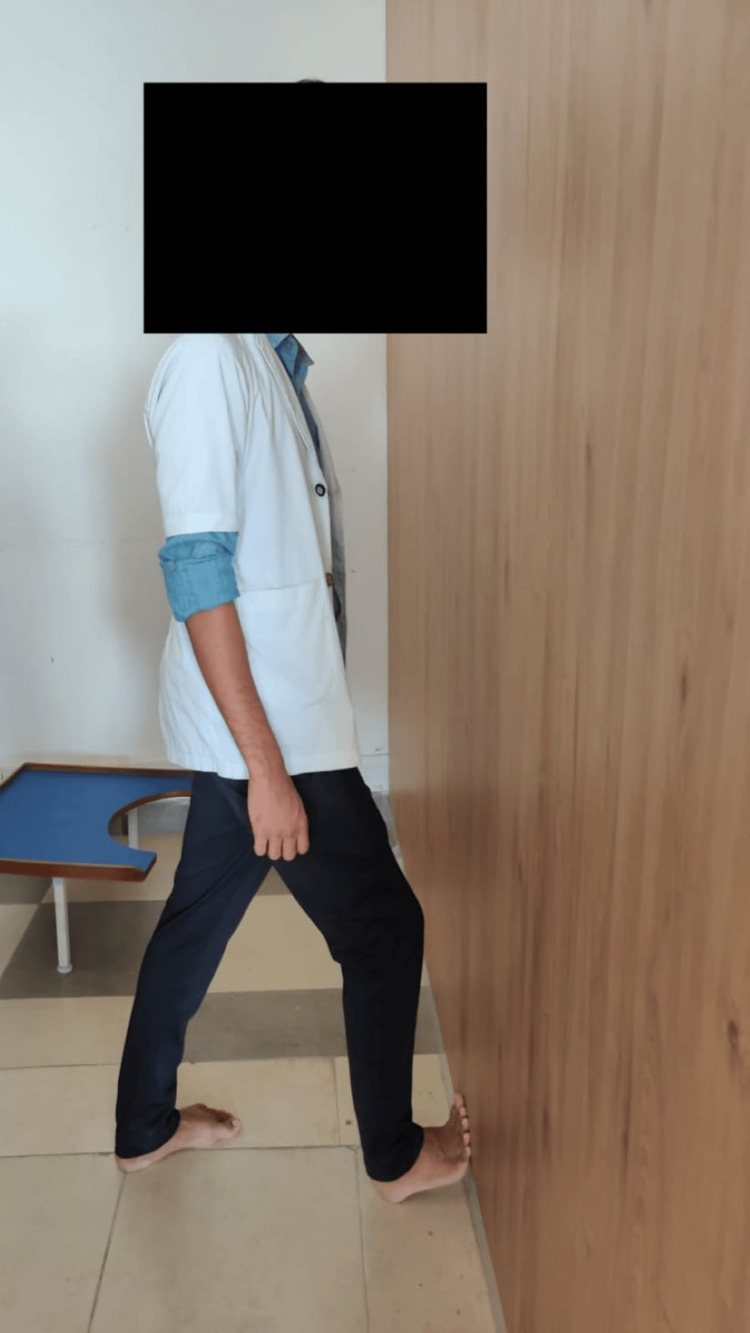
Heel cord stretching exercises Begin by standing facing a wall with one leg forward and slightly bent at the knee. Encourage participants to straighten the other leg behind them, ensuring both heels are flat on the ground. Instruct them to gently press their hips towards the wall, maintaining this position for 30 seconds before relaxing for another 30 seconds. Repeat this sequence 10 times.

**Figure 4:**
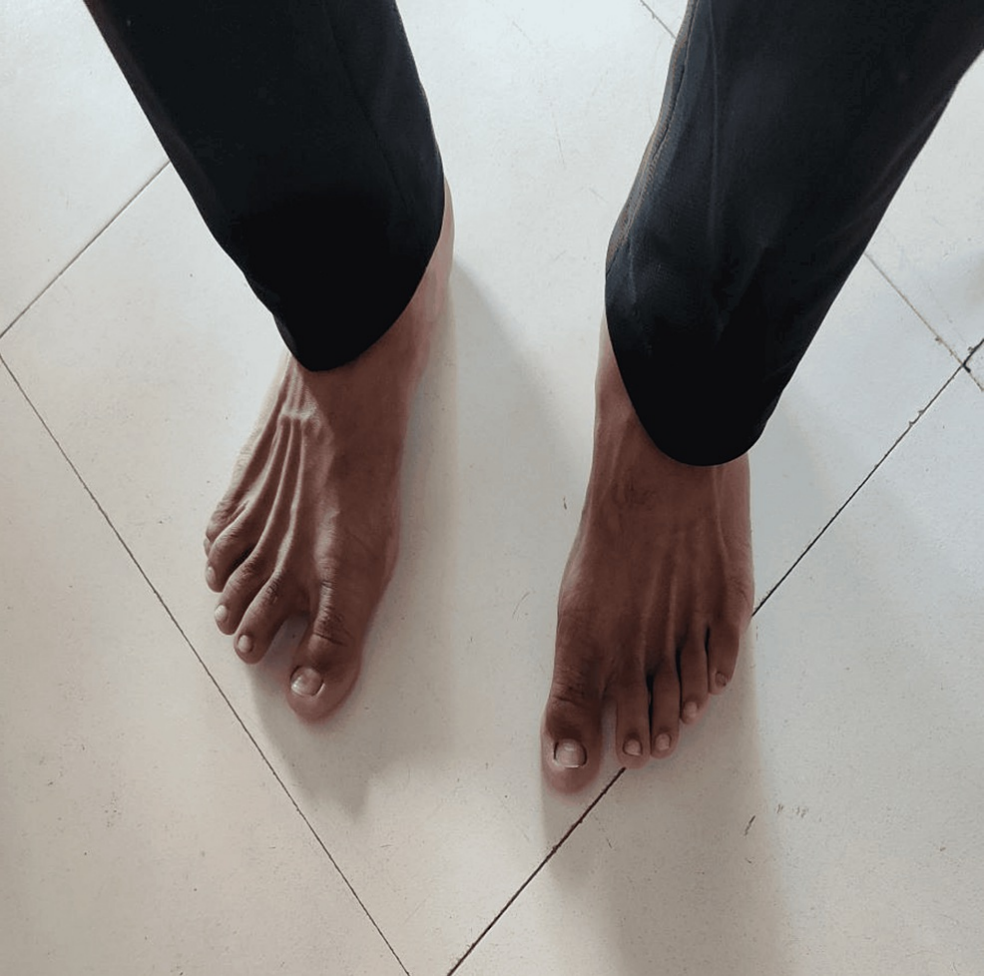
Toe spread exercise While seated on a chair with feet flat on the floor, prompt individuals to spread their toes as wide apart as possible. Hold this position for five seconds, then relax for two seconds before repeating.

Furthermore, specific exercises such as toe spreading and posterior tibialis exercises further enhance therapeutic outcomes. Toe spreading, accomplished by sitting on a chair and spreading toes apart for brief intervals, promotes foot stability and toe dexterity. Meanwhile, posterior tibialis exercises, executed while seated with resistance bands, target specific muscles crucial for ankle stability and function. These exercises (Table 1, Figures 2-4) performed with prescribed sets and repetitions over a designated period, contribute to comprehensive physiotherapy management, facilitating rehabilitation, and promoting overall physical well-being.

**Table 1:** Foot strengthening exercises

SL NO	Intervention	Procedure	Intensity
1	Towel scrunching exercise (Figure 2)	Instruct the individual to engage in the towel-gathering activity by scrunching a towel placed on the floor with their toes	15 minutes daily
2	Heel cord stretching (Figure 3)	Begin by standing facing a wall with one leg forward and slightly bent at the knee. Encourage participants to straighten the other leg behind them, ensuring both heels are flat on the ground. Instruct them to gently press their hips toward the wall.	Maintaining this position for 30 seconds before relaxing for another 30 seconds. Repeat this sequence 10 times.
3	Toe spread exercise (Figure 4)	While seated on a chair with feet flat on the floor, prompt individuals to spread their toes as wide apart as possible.	Hold this position for 5 seconds, then relax for 2 seconds before repeating.
4	Seated posterior tibialis exercises	Instruct participants to sit with crossed legs and secure a resistance band around one foot, with the other end anchored under the opposite foot. Keeping the ankle relaxed, guide them to move the foot upwards toward the ceiling, then slowly return to the starting position.	Perform 3 sets of 10 repetitions for each foot, scheduling this exercise routine once a week for 60 days.

Outcome measures

The outcome measures used were manual muscle testing, a visual analog scale, a foot health status questionnaire, and an arch index. The outcomes are assessed twice, that is before the treatment (baseline) and after four weeks of intervention (post-intervention) which were mentioned in Table 2.

**Table 2:** Pre- and post-outcome measures Visual analog scale: 0: no pain; 10: worst pain; Foot health status questionnaire: 0: worst foot health; 100: good foot health; Arch index: less than or equal to 0.21 is a high arch; more than or equal to 0.26 is a high arch.

SL NO	Outcome measures	Case 1 pre-treatment (/n)	Case 1 post-treatment (/n)	Case 2 pre-treatment (/n)	Case 2 post-treatment (/n)
1	Manual muscle strength (MMT) of foot intrinsic muscles	Grade 3/Grade 5	Grade 5/Grade 5	Grade 2/Grade 5	Grade 5/Grade 5
2	Visual analog scale (VAS)	4/10	0/10	6/10	0/10
3	Foot health status questionnaire (FHSQ)	40/100	90/100	60100	80/100
4	Arch index	0.26	0.26	0.21	0.21

## Discussion

The effect of foot rehabilitation protocols on adults with congenital foot deformities represents a critical area of study aimed at improving their functional abilities and overall quality of life. This case series examines the impact of a comprehensive foot rehabilitation protocol on two cases of adults with congenital foot deformities, offering insights into the efficacy and potential benefits of such interventions. One significant component of the rehabilitation protocol employed in this study is the towel-gathering exercise, where participants are instructed to scrunch a towel lying on the floor with their toes for 15 minutes daily. This exercise targets intrinsic foot muscles, aiming to improve strength, flexibility, and dexterity in the feet [11-15].

Furthermore, the protocol includes heel cord stretching exercises, designed to address issues such as tightness and limited range of motion in the Achilles tendon and calf muscles. Participants are guided to stand facing a wall with one leg forward and the other leg straight behind, ensuring both heels are flat on the ground. By gently pressing their hips toward the wall and holding the stretch for 30 seconds before relaxing, participants work toward improving flexibility and reducing tension in the lower limbs. This exercise, repeated for 10 sets, aims to optimize joint alignment and mobility. In addition to the aforementioned exercises, the rehabilitation protocol incorporates toe-spreading activities. Participants are seated on a chair with both feet flat on the floor and instructed to spread their toes as far apart as possible for five seconds, followed by a two-second relaxation period [16,17]. This exercise targets toe mobility and foot stability, aiming to address issues related to toe deformities and improve overall foot function.

Moreover, the rehabilitation protocol includes posterior tibialis exercises performed in a seated position. Participants cross their legs and tie a resistance band around one foot, with the other end anchored under the opposite foot. By moving the foot upwards towards the ceiling against resistance and returning to the starting position slowly, participants engage the posterior tibialis muscle, which plays a crucial role in foot and ankle stability. This exercise regimen, comprising 3 sets of 10 repetitions, is performed five days a week for 60 days, with the goal of enhancing muscle strength and coordination [18-20].

Furthermore, inherent biases, such as selection bias and observer bias, may affect the validity of the results. The lack of long-term follow-up limits understanding regarding the sustainability of the effects over time. Additionally, reliance on subjective outcome measures and potential ethical considerations could introduce variability and raise concerns about the study's integrity. Thus, while the findings provide valuable insights, caution should be exercised in extrapolating them to wider populations, and further research with robust study designs is warranted to validate the efficacy of the foot rehabilitation protocol. Additionally, further studies should prioritize larger sample sizes, incorporate control groups for comparison, utilize objective outcome measures such as biomechanical assessments or radiographic imaging, implement longer follow-up periods to assess sustained improvements, and include diverse populations to enhance the generalizability of the findings across different demographics and geographical regions.

## Conclusions

In conclusion, the case series exploring the effect of a foot rehabilitation protocol on adults with congenital foot deformities demonstrates promising outcomes in improving functional abilities and enhancing the quality of life for affected individuals. Through a comprehensive regimen that includes exercises targeting muscle strength, flexibility, and coordination, along with therapeutic interventions tailored to address specific impairments, significant improvements were observed in the participant's overall foot function and mobility. The inclusion of exercises such as towel gathering, heel cord stretching, toe spreading, and posterior tibialis exercises proved in this case are beneficial in addressing various aspects of foot deformities, including muscle weakness, joint stiffness, and toe deformities. By implementing these exercises regularly over a period of 60 days, participants exhibited enhanced muscle strength, improved joint mobility, and greater stability in the feet and ankles.
